# Evidence for modulation of the fecal microbiota profile by diet in lactating buffalo

**DOI:** 10.3389/fvets.2026.1739986

**Published:** 2026-02-17

**Authors:** Chiara Evangelista, Daniele Pietrucci, Marco Milanesi, Federica Gabbianelli, Loredana Basiricò, Sebastiana Failla, Giovanni Chillemi, Umberto Bernabucci

**Affiliations:** 1 Department for Innovation in Biological Agro-Food and Forest Systems, University of Tuscia, Viterbo, Italy; 2 Department of Agriculture and Forests Sciences, University of Tuscia, Viterbo, Italy; 3 Department of Animal Sciences, Food and Nutrition (DIANA), Università Cattolica del Sacro Cuore, Piacenza, Italy; 4 CREA-Research Center for Animal Production and Aquaculture, Monterotondo, Italy; 5 Department of Experimental Medicine, University of Rome “Tor Vergata”, Rome, Italy

**Keywords:** dairy buffaloes, fecal microbiota, feeding, microbial diversity, next-generation sequencing

## Abstract

**Introduction:**

The gastrointestinal microbiota of ruminants plays a crucial role in health, influencing immune responses, nutrient metabolism, and environmental impact. While the ruminal microbiota has been widely investigated, the hindgut microbiota, particularly the fecal microbiota, remains less explored. Diet strongly shapes microbial communities, thereby affecting digestion, metabolic pathways, and methane emissions. Next-generation sequencing enables detailed microbiota profiling; however, no studies have characterized the fecal microbiota of Italian Mediterranean buffaloes in relation to diet. This study aimed to evaluate the bacterial composition and dietary influences on the fecal microbiota of dairy buffaloes.

**Methods:**

Over 6 months, monthly pooled fecal samples were collected from ~10 to 15% of lactating buffaloes across 10 farms. Concurrently, dietary data were recorded, and total mixed ration samples were analyzed for physicochemical properties and fatty acid profiles. DNA was extracted using the Quick-DNA™ kit, followed by 16S rRNA sequencing on an Illumina MiSeq System. Statistical analyses in R included alpha and beta diversity, differential abundance testing, and one-way ANOVA (*p* < 0.05; trends at *p* < 0.10).

**Results:**

In total, 10 phyla, 13 classes, 26 orders, 47 families, 86 genera, and 120 species were identified. *Firmicutes* was the most abundant phylum (55.8 ± 3.6%), followed by *Bacteroidota* (37.7 ± 3.4%). Among dietary variables, the forage-to-concentrate (FC) ratio and linseed (LS) inclusion exerted the greatest influence. Notably, the FC ratio affected beta diversity (community structure) but not alpha diversity (within-sample diversity), whereas LS inclusion influenced both alpha and beta diversity. A low FC ratio promoted phyla negatively associated with fiber digestibility, particularly families *Lachnospiraceae* and *Succinivibrionaceae*, consistent with cattle studies linking these taxa to high-concentrate diets rich in fine particles (<4 mm). Linseed inclusion reduced species richness and increased *Firmicutes, Spirochaetota*, and *Proteobacteria*, the latter including potential pathogens implicated in ruminal dysbiosis. Conversely, LS inclusion decreased *Verrucomicrobiota*, a phylum important for gut health and mucus layer maintenance.

**Conclusion:**

This study provides the first characterization of the fecal microbiota of Italian Mediterranean dairy buffaloes and highlights its responsiveness to diet. Findings underscore the potential of fecal microbiota as a non-invasive biomarker for evaluating dietary effects, with implications for animal health, productivity, and environmental sustainability.

## 1 Introduction

Dairy ruminant farming represents a cornerstone of the global agricultural economy, providing a vital source of dairy products that play a key role in human nutrition worldwide. Considering the growing demand for more sustainable and efficient agricultural systems, a comprehensive understanding of the ruminal microbial ecosystem has become essential to optimize livestock health, enhance productivity, and mitigate the environmental impact of dairy farming. The gastrointestinal tract (GIT) microbiome of ruminants constitutes a highly intricate and dynamic ecosystem, where the host, microbiota, and environmental factors interact in complex ways to shape animal health and performance. Maintaining gut health is fundamental to preventing infectious diseases, as intestinal homeostasis and immune system balance are pivotal to overall wellbeing. A well-functioning microbiota plays a central role in modulating both local (mucosal) and systemic immune responses, providing protection against pathogen colonization and supporting host defenses (1, 2). Beyond immune modulation, the gut microbiota is essential for the synthesis of key nutrients—including amino acids, fatty acids, and vitamins—which contribute to metabolic regulation, energy homeostasis, and immune function (3).

The composition of microbial communities within GIT is influenced by a wide range of factors, including species, breed, age, nutrition, environmental conditions, rearing practices, stocking density, stress, antibiotic usage and reproductive status, such as oestrus (1, 3, 64). Some authors have reported that the rumen and feces share a bacterial “core microbiome” at the family and/or genus level, which plays a pivotal role in the GIT ecosystem (4). This overlap highlights the interconnectedness and mutual influence between these microbial communities (4). Given the growing interest in less invasive sampling methods to associate microbial populations with host phenotypes and functional traits (5), the fecal microbiota has gained prominence as a valuable area of investigation. Highly responsive to dietary changes (6), the fecal microbiota is primarily shaped by the animal's diet, with the forage-to-concentrate (FC) ratio recognized as a key driver of its composition and diversity (3, 6, 7). Understanding how dietary factors influence rumen vs. fecal microbiota is essential to interpret fecal profiles in the context of gut health and animal performance.

Ruminant nutrition significantly impacts the ruminal microbiome, which in turn influences the fecal microbiome. The ruminant diet primarily consists of plant-derived materials that undergo fermentation in the rumen. This fermentation process produces volatile fatty acids, carbon dioxide (CO_2_), and methane (CH_4_). Additionally, the rectum functions as a complementary digestive organ by utilizing undigested but potentially digestible substrates to produce short-chain fatty acids (SCFA), a vital energy source for the host (8), with its role becoming particularly significant when transit rates are high or ruminal function is impaired, leading to an increased flow of substrates escaping ruminal degradation (9). Diets low in forage and rich in degradable carbohydrates promote the growth of amylolytic bacteria, reducing CH_4_ production (10). However, such diets increase the risk of ruminal acidosis, a metabolic disorder that can impair overall health and productivity (6). On the other hand, high-fiber diets—while less efficient for energy production—generate higher CH_4_ emissions per kilogram of milk produced (10).

These dietary interactions underscore the intricate balance between optimizing energy production and mitigating the environmental impacts of ruminant systems. Understanding how nutrition influences microbial communities is essential for developing strategies that enhance animal health, productivity, and sustainability. In this context, studying the fecal microbiota offers a valuable approach not only for gaining insights into feed efficiency but also for monitoring CH_4_ emissions and assessing animal welfare in a non-invasive manner (11, 12).

Despite extensive research on the ruminal microbiota, the hind-gut microbiome, represented by the fecal microbiota, remains less characterized particularly through advanced techniques like next-generation sequencing (NGS) (13). Next-generation sequencing is currently regarded as the most reliable method for assessing bacterial diversity in both the rumen and feces of large ruminants [e.g., cattle, Cendron et al. (11)]. The 16S rRNA gene is commonly utilized as a reference for determining the composition of bacterial communities due to its phylogenetic variability (11).

To date, no studies have characterized the microbiota composition of Italian Mediterranean dairy buffalo feces in relation to diet supplied, nor have any utilized NGS for this purpose. Therefore, the objectives were twofold: to characterize the bacterial composition of dairy buffalo feces using NGS technique and to evaluate the influence of diet supplied on bacterial communities and their effect on animal performance. By integrating these objectives with existing literature on ruminal and fecal microbiota, this study aims to provide preliminary insights into the diet-microbiota relationships in dairy buffalo, which may inform future research on nutritional management and sustainability in ruminant production.

## 2 Materials and methods

### 2.1 Animal ethics

No approval of research ethics committees was required to accomplish the goals of this study because experimental work was conducted within the normal procedures for handling animals adopted by dairy buffalo farms hosting the trial. All applicable international, national, and/or institutional guidelines for the care and use of animals were followed. The procedure of fecal samples collection was carried out by the veterinary following the routine procedure and out of the scope of Directive 2010/63/EU (art. 1.5.f “practices not likely to cause pain, suffering, distress or lasting harm equivalent to, or higher than, that caused by the introduction of a needle in accordance with good veterinary practice”).

### 2.2 Experimental design

Ten dairy buffalo farms representative of the Amaseno Valley area (Lazio, Italy) were selected and monitored consecutively over 6 months, from June to November 2022. Each month, samples of total mixed ration (TMR) and group feces were collected, along with information on the composition of the TMR.

### 2.3 Sampling procedure

The TMR were collected immediately after morning distribution, an aliquot was used for physical characterization, another aliquot was stored at 4 °C for laboratory chemical analyses. Fecal samples were collected approximately 2–3 h after the distribution of the morning feeding. Sampling was conducted on a representative group of animals, consisting of approximately 8–10 lactating buffaloes per farm, with days in milk ranging from 0 to 240. This group represented about 10%−15% of the total lactating buffalo population on each farm, in accordance with the guidelines proposed by Pennsylvania Management (14). Samples were obtained directly from the rectal ampoule to ensure accuracy and consistency. Buffaloes were visibly healthy, and no illnesses among these animals were reported after sample collection. The individual fecal samples were then combined and homogenized to create a composite sample, referred to as “group feces.” Then, the pH was immediately determined on the fresh group feces using a portable pH meter (XS Tester, Giorgio Bormac S.r.l., Carpi, Italy) which was calibrated using standard solutions of pH 4 and 7. An aliquot of feces was stored at 4 °C for subsequent chemical analysis, while another aliquot was stored in triplicate using DNA/RNA Shield™ Lysis Tubes (Zymo Research, Irvine, CA, USA), which contain a liquid preservative designed to maintain sample integrity during storage. The samples were refrigerated at 4 °C for a maximum of 24 h before being stored at −80 °C as soon as possible, as recommended by Jaramillo-Jaramillo et al. (2), until DNA extraction. In total, 60 TMR and 60 group faces samples were collected.

### 2.4 Sampling analysis

The particle size distribution of the TMR was determined as-fed using the Penn State Particle Separator (PSPS), following the procedure outlined by Heinrichs (15) and the methods described by Evangelista et al. (16). The PSPS separates the sample into four fractions: Upper Sieve (US, >19 mm), Medium Sieve (MS, 8–19 mm), Lower Sieve (LS, 4–8 mm), and Bottom (<4 mm). Each fraction was weighed, and its percentage relative to the total sample weight was calculated.

Both TMR and fecal samples underwent chemical analysis. Dry matter (DM) was determined by oven-drying at 65 °C to constant weight. Samples were then ground using a mill (Retsch GmbH, Haan, Germany) to pass through a 1 mm screen and stored in sealed polyethylene containers. Analyses included crude protein (CP) (17), ash (18), ether extract (EE) (19), and starch (17), the latter determined using a K-TSTA assay kit (Megazyme International, Bray, Ireland). Crude fiber (CF) (17), neutral detergent fiber (aNDF), acid detergent fiber (ADF), and acid detergent lignin (ADL) were analyzed using an Ankom200 Fiber Analyzer (Ankom Technology, Macedon, NY), according to Van Soest et al. (20). All chemical data are expressed as percentages on a dry matter basis.

Fatty acid (FA) analysis of TMR was performed using 10 g of dried TMR, following the method described by Folch et al. (21) with a chloroform mixture (2:1, v/v). The fat was dissolved in hexane, methylated with methanolic KOH, and a 1 ml sample was injected into a gas chromatograph with a flame ionization detector (GC-FID; Agilent Technologies, Santa Clara, CA, USA), equipped with a CP-Sil88 capillary column (100 m, 0.25 mm, 0.20 μm, 100% cyanopropyl). C19:0 was used as the internal standard. GC-FID conditions included a splitless inlet at 250 °C, FID at 250 °C, and helium carrier gas at 1.2 ml min^−1^. The temperature program started at 60 °C (held for 4 min) and ramped up to 175 °C (held for 27 min), then to 215 °C (held for 25 min), and finally to 220 °C (held for 5 min). Fatty acid peaks were identified by comparison with a Supelco Mix 37 standard (Sigma-Aldrich Merck), which includes common fatty acids such as C14:0, C16:0, C18:0, C18:1 cis-9, and others. Fatty acid methyl esters (FAME) were expressed as a percentage of total FAME content. The total amounts of saturated fatty acids (SFA), monounsaturated fatty acids (MUFA), and polyunsaturated fatty acids omega-6 (PUFA n-6) and omega-3 (PUFA n-3) were also calculated.

Physically effective NDF (peNDF) was calculated as follows (22):


$$\begin{array}{l} peNDF\;=\;\left[US\;\left(\%\right)\;+\;MS\;\left(\%\right)\;+\;LS\;\left(\%\right)\right]\;*\;aNDF\;\left(\%\right) \end{array}$$

where US, MS, and LS are the proportion of particles retained by the 19.0, 8.0, and the 4.0 mm screen, respectively.

Apparent digestibility of aNDF, starch, CP, EE, and DM was estimated using ADL as internal marker (23):


$$\begin{array}{l} aNDF,starch,CP,EE\;\left(\%\right)=\{1-[\frac{ADL\;TMR}{ADL\;faeces}\\ \;\times \;\frac{Analyte\;faeces}{Analyte\;TMR}]\}\times \;100\\ \;\;DM\;\left(\%\right)=\left\{\left[\frac{ADL\;faeces-ADL\;TMR}{ADL\;faeces\;}\right]\right\}\times \;100\; \end{array}$$

### 2.5 Sample selection

Of the 60 collected group fecal samples, a selection process was implemented to identify and exclude those with similar characteristics regarding diet composition, in order to avoid including duplicate samples with the same diet composition (e.g., samples collected over two consecutive months in which the diet was not changed). This was done to ensure a diverse representation of the fecal microbiota composition. The selection process involved detailed data analysis and visualization techniques. First, a heatmap (Supplementary Figure S1) was used to display the similarity between samples based on their chemical composition. Next, a dendrogram (Supplementary Figure S2) grouped the samples by similarity, revealing that adjacent samples and those on the same branches were more alike. Through these analyses, 13 samples, identified as highly similar to others, were subsequently excluded from DNA extraction, resulting in 47 fecal samples considered for further DNA extraction.

### 2.6 Fecal DNA extraction

DNA was extracted from fecal samples using the Quick-DNA™ Fecal/Soil Microbe Miniprep Kit (Zymo Research Corporation, Irvine, CA, USA), following the manufacturer's protocol with minor modifications: 300 mg of sample was used instead of the recommended 150 mg. DNA quantification was performed using a DTX Multimode Detector 880 (Beckman Coulter, Vienna, Austria) with the Quant-iT™ PicoGreen™ dsDNA Assay Kits (Invitrogen by Thermo Fisher Scientific, Eugene, Oregon, USA), following the manufacturer's instructions. After quantification, another six samples were removed due to low extraction yield, inadequate for further analysis. None of them had a sample removed in the previous analyses. As a result, a total of 41 samples were retained for downstream analysis.

16S metagenomic sequencing libraries were prepared using the 16S V3-V5 Library Preparation Kit for Illumina (Norgen Biotek Corp., Thorold, Ontario, Canada), according to the manufacturer's guidelines, which ensures coverage of the entire V3-V4 region. Input DNA underwent targeted PCR to amplify the V3–V5 region of the 16S rRNA-encoding DNA. The resulting PCR products were cleaned using Mag-Bind Total Pure NGS beads (Omega Bio-Tek, Norcross, Georgia, USA). Dual index primers were added during a limited-cycle PCR to generate indexed amplicons flanked by 5′ and 3′ barcoded adaptors. These amplicons were subsequently cleaned using Mag-Bind Total Pure NGS beads.

The libraries were quantified using a DTX Multimode Detector 880 with the PicoGreen method yielding an average DNA concentration of 25 ng μl^−1^. Libraries were diluted to a concentration of 4 nM, denatured to 8 pM, and pooled using the MiSeq Reagent Kit v3 (600-cycle; Illumina, San Diego, CA, USA). The pooled libraries were loaded onto the flow cell and sequenced on a MiSeq System.

### 2.7 Bioinformatics and statistical analyses

The quality of raw sequencing data was assessed using FastQC (24), followed by primer and adapter sequence trimming with Cutadapt (25). Processed reads were analyzed using the QIIME 2 pipeline (26), incorporating chimera detection and clustering into amplicon sequence variants (ASV) via the DEBLUR algorithm with default settings (27). Taxonomic classification of representative ASVs generated by DEBLUR was performed using the q2-feature-classifier plugin (28), with the Greengenes2 database version 2022.10 (29). Statistical analyses were conducted in the R 4.3.0 environment using the vegan version 2.6.4 (30) and phyloseq version 1.46.0 (31) packages. Low-abundance ASV, defined as those contributing less than 0.005% of total reads across samples (32), were excluded. The remaining sequences were normalized via rarefaction, setting the seed to 1 and selecting an optimal sample size based on rarefaction curves generated with the ggrare function of ranacapa package version 0.1.0. Alpha diversity was quantified using the number of Observed Species, Shannon, Simpson, and Fisher indices, with statistical significance assessed via the Wilcoxon–Mann–Withney exact test. To increase the robustness of statistical inference given the small and unbalanced group sizes, permutation-based *p*-values were additionally estimated using a Monte Carlo resampling approach (10,000 permutations). Beta diversity metrics, including Bray-Curtis distances and weighted generalized UniFrac, were calculated, and significance was tested using the Permutational Multivariate Analysis of Variance (PERMANOVA) method with 9,999 permutations. Differentially abundant taxa were identified using the DESeq2 algorithm applied to the unrarefied dataset (33, 34). Data visualization, including relative abundance plots, boxplots for alpha diversity, and principal coordinates analysis for beta diversity, was performed using the ggplot2 package (35).

Based on previous studies carried out on dairy cows and goats (3, 6, 36), dietary and management variables potentially influencing the microbiota were selected from the available metadata (Supplementary Table S1). Differences in bacterial community composition among variables were assessed using PERMANOVA. To facilitate group comparisons, variables were categorized as high (H), medium (M), or low (L), or as binary variables (yes/no) when appropriate (e.g., linseed inclusion, green forage presence). Pairwise comparisons were performed between extreme categories (H vs. L or yes vs. no) to enhance the detection of community-level differences. Variables showing strong collinearity were excluded, and subsequent analyses focused on two key dietary factors: forage-to-concentrate ratio (FC_H vs. FC_L) and linseed inclusion (LS_no vs. LS_yes), which captured the main sources of dietary variation.

Group differences between FC_H and FC_L (*n* = 13 per group) and between LS_no (*n* = 30) and LS_yes (*n* = 11) were subsequently evaluated using one-way analysis of variance (ANOVA). *P*-values between 0.05 and 0.10 were reported as indicative of trends and were interpreted as exploratory signals rather than definitive effects.

## 3 Results

### 3.1 Diet and feces composition

The composition of the diets subdivided based on the four groups: FC high and low (FC_H and FC_L, respectively) and presence or not of LS (LS_yes and LS_no, respectively), are listed in Table 1. The FC_L and FC_H diets had FC ratio of 65:35 and 85:15, respectively. For the LS_yes a linseed-based mixture (Linomix, Table 1), was used at a dosage of 0.5 kg per head per day, representing approximately 1.66% of the total diet on an as-fed basis.

**Table 1:** Distribution of feed ingredients in the diets (mean ± SD).

**Components % on as it is**	**FC_H (*n.13*)**	**FC_L (*n.13*)**	**LS_no (*n.30*)**	**LS_yes (*n.11*)**
Silages	62.80 ± 6.92	41.36 ± 6.93	51.74 ± 8.14	43.90 ± 15.36
Hays	22.24 ± 6.87	17.75 ± 4.76	22.20 ± 8.79	25.40 ± 6.05
Green fodder	–	14.59 ± 5.14	8.40 ± 2.61	17.80 ± 4.04
Threshers	–	20.68 ± 4.55	20.65 ± 4.49	17.19 ± 3.83
Concentrates	14.23 ± 3.42	13.46 ± 1.87	19.21 ± 3.83	14.59 ± 3.53
Linomix mixture^a^	1.65 ± 0.10	1.62 ± 0.07	–	1.66 ± 0.33
Supplements	0.76 ± 0.61	0.85 ± 0.54	0.98 ± 1.07	0.44 ± 0.07
FC ratio	85:15	65:35	76:24	74:26

FC_H, high forage-to-concentrate ratio; FC_L, low forage-to-concentrate ratio; LS_no, diets without linseed; LS_yes, diets with linseed; The silages used included corn and triticale; Hay consisted of oat, alfalfa, straw, clover, and ryegrass; Green forage was composed of sorghum (Sorghum bicolor) and/or Italian ryegrass (Lolium italicum); the concentrate portion included corn flour, barley flour, soybean meal, barley radicles, cottonseeds, and horse bean seeds; Supplements consisted of yeasts, vitamins, calcium salts of palm fatty acids, and calcium carbonate (CaCO_3_).

^a^Linomix mixture: extruded lineseeds, sugar beet pulp, corn, sodium chloride, calcium carbonate, wheat. Nutritional analysis: crude protein 16%, crude fat 20%, crude fiber 13%, crude ash 8.5%, sodium 0.52%.

The physicochemical composition of the diets is presented in Table 2. The FC ratio had a greater influence in modifying the diet compared to the presence of LS, significantly impacting DM (*p* < 0.01), ash (*p* < 0.05), CP (*p* < 0.01), bottom (*p* < 0.01), and milk forage unit (MFU; *p* < 0.05). In contrast, LS had, as expected, a greater influence on fatty acid composition of the TMR (Table 3). The presence of LS lead to a reduction (*p* < 0.01) of C14:0, C16:0, C16:1, C18:2n6c (LA), SFA, PUFAn6, and the n6/n3 ratio, and simultaneously increased (*p* < 0.01) C18:3n3 (ALA), PUFAn3, and PUFAtot in TMR. Conversely, FC_H ratio reduced (*p* < 0.05) C20:1n9 and increased (*p* < 0.05) C24:0 and C20:0.

**Table 2:** Physicochemical composition of total mixed rations, divided into four dietary groups (LSMeans ± SE).

**Component**	**FC_H (*n.13*)**	**FC_L (*n.13*)**	**LS_no (*n.30*)**	**LS_yes (*n.11*)**
Dry matter, %	54.57 ± 1.46^A^	46.82 ± 1.43^B^	52.19 ± 1.06	52.71 ± 2.28
Ashes, % DM^−1^	6.93 ± 0.20	6.65 ± 0.20	7.09 ± 0.17^a^	6.27 ± 0.17^b^
Crude protein, % DM^−1^	11.12 ± 0.46^A^	12.46 ± 0.32^B^	11.98 ± 0.39	12.23 ± 0.32
Ether extract, % DM^−1^	2.28 ± 0.09	2.42 ± 0.11	2.53 ± 0.09	2.47 ± 0.07
Crude fiber, % DM^−1^	28.84 ± 1.88	29.60 ± 1.92	29.02 ± 1.15	26.24 ± 1.87
aNDF, % DM^−1^	48.28 ± 1.21	47.97 ± 1.12	46.94 ± 1.04	44.53 ± 1.07
ADF, % DM^−1^	32.68 ± 0.84	31.17 ± 0.72	31.42 ± 0.78	30.32 ± 0.98
ADL, % DM^−1^	6.31 ± 0.29	6.14 ± 0.18	6.27 ± 0.19	5.96 ± 0.32
Starch, % DM^−1^	13.11 ± 0.68	12.76 ± 0.70	13.03 ± 0.49	14.42 ± 0.81
Upper Sieve, %	25.22 ± 5.67	22.93 ± 3.95	20.09 ± 3.12	21.39 ± 3.75
Middle Sieve, %	27.88 ± 3.47	20.79 ± 2.02	24.52 ± 1.72	25.72 ± 2.98
Lower Sieve, %	26.04 ± 2.59	29.40 ± 1.76	30.33 ± 1.79	25.75 ± 1.96
Bottom, %	20.84 ± 1.44^A^	27.29 ± 1.83^B^	24.41 ± 1.22	27.14 ± 1.63
peNDF, %	38.36 ± 1.55	35.19 ± 1.44	35.81 ± 1.24	32.47 ± 1.21
MFU, kg as feed	0.82 ± 0.02^b^	0.87 ± 0.01^a^	0.85 ± 0.01	0.88 ± 0.01

FC_H, high forage-to-concentrate ratio (85:15); FC_L, low forage-to-concentrate ratio (65:35); LS_no, diets without linseed; LS_yes, diets with linseed; DM, dry matter; aNDF, neutral detergent fiber; ADF, acid detergent fiber; ADL, lignin acid deterse; Upper Sieve=particles >19 mm; Middle Sieve=particles >8mm; Lower Sieve=particles >4 mm; Bottom=particles <4mm; MFU, milk forage unit. Pairwise comparisons: FC_H vs FC_L; LS_no vs. LS_yes; ^a, b^ = *p* < 0.05; ^A, B^ = *p* < 0.01.

**Table 3:** Fatty acid composition of total mixed rations, divided into four dietary groups (Lsmean ± SE).

**Fatty acid, g·100^−^1 g**	**FC_H (*n.13*)**	**FC_L (*n.13*)**	**LS_no (*n.30*)**	**LS_yes (*n.11*)**
C14:0	0.47 ± 0.03	0.45 ± 0.04	0.50 ± 0.02^A^	0.35 ± 0.02^B^
C16:0	16.43 ± 0.63	17.81 ± 0.59	18.12 ± 0.30^A^	14.78 ± 0.50^B^
C16:1	0.40 ± 0.04	0.47 ± 0.04	0.46 ± 0.02^A^	0.36 ± 0.04^B^
C18:0	3.25 ± 0.13	2.94 ± 0.15	2.89 ± 0.09	3.27 ± 0.08
C18:1n9/cis9	20.91 ± 0.46	19.37 ± 0.75	20.29 ± 0.41	20.76 ± 0.61
C18:1n7/cis11	1.07 ± 0.02	0.99 ± 0.02	1.00 ± 0.02	1.04 ± 0.02
C18:2n6c LA	43.03 ± 0.75	42.43 ± 1.28	45.45 ± 0.59^A^	39.04 ± 0.88^B^
C20:0	0.54 ± 0.02^a^	0.48 ± 0.02^b^	0.50 ± 0.02	0.49 ± 0.02
C18:3n6 GLA	2.03 ± 0.15	1.82 ± 0.16	1.82 ± 0.12	1.63 ± 0.17
C18:3n3 ALA	9.98 ± 1.31	11.11 ± 1.65	7.15 ± 0.57^B^	16.40 ± 1.05^A^
C20:1n9	0.41 ± 0.03^b^	0.63 ± 0.04^a^	0.44 ± 0.02	0.59 ± 0.07
C20:3 n6	0.38 ± 0.01	0.37 ± 0.01	0.36 ± 0.01	0.34 ± 0.01
C20:4 n6 AA	0.74 ± 0.10	0.78 ± 0.14	0.66 ± 0.07	0.63 ± 0.15
C20:5n3	0.02 ± 0.003	0.02 ± 0.003	0.02 ± 0.002	0.02 ± 0.002
C24:0	0.36 ± 0.01^a^	0.35 ± 0.01^b^	0.34 ± 0.01	0.34 ± 0.01
SFA	21.07 ± 0.69	22.02 ± 0.54	22.35 ± 0.31^A^	19.23 ± 0.48^B^
MUFA	22.76 ± 0.44	21.46 ± 0.77	22.20 ± 0.42	22.71 ± 0.57
PUFAn6	46.18 ± 0.89	45.40 ± 1.29	48.28 ± 0.50^A^	41.65 ± 0.88^B^
PUFAn3	10.00 ± 1.31	11.12 ± 1.65	7.17 ± 0.57^B^	16.41 ± 1.05^A^
PUFAtot	56.18 ± 0.50	56.52 ± 0.83	55.45 ± 0.36^B^	58.06 ± 0.41^A^
n6/n3	5.49 ± 0.59	5.41 ± 0.79	7.80 ± 0.57^A^	2.67 ± 0.21^B^

FC_H, high forage-to-concentrate ratio (85:15); FC_L, low forage-to-concentrate ratio (65:35); LS_no, diets without linseed; LS_yes, diets with linseed; C14:0, myristic; C16:0, palmitic; C16:1, palmitoleic; C18:0, stearic; C18:1n9/cis9, oleic; C18:1n7/cis11, vaccenic; C18:2n6c LA, linoleic; C20:0, arachidic; C18:3n6 GLA, gamma-linolenic; C18:3n3 ALA, alpha-linolenic; C20:1n9, gadoleic; C20:3 n6, dihomo-gamma-linolenic; C20:4 n6 AA, arachidonic; C20:5n3, eicosapentaenoic (EPA); C24:0, lignoceric; SFA, saturated fatty acids; MUFA, monounsaturated fatty acids; PUFAn6, polyunsaturated fatty acids omega-6; PUFAn3, polyunsaturated fatty acids omega-3; PUFAtot, total polyunsaturated fatty acids; n6/n3, Omega-6/Omega-3 ratio. Pairwise comparisons: FC_H vs. FC_L; LS_no vs. LS_yes; ^a, b^*p* < 0.05; ^A, B^*p* < 0.01.

The composition of the group feces obtained from the different diets shows differences in nearly all the analyzed parameters, except for ash content, which does not differ among any of the groups (Table 4). The FC ratio influenced EE (*p* < 0.01), which was higher in FC_L, while ADF (*p* < 0.01), ADL (*p* < 0.01), and pH (*p* < 0.01), were lower in FC_L. The presence of LS was the factor that had the most influence on the chemical composition of the feces. Its presence (LS yes) increased DM content (*p* < 0.01), CP (*p* < 0.05), EE (*p* < 0.01), and starch (*p* < 0.01), while reducing aNDF (*p* < 0.05), ADF (*p* < 0.01), and pH (*p* < 0.01). In contrast, the FC ratio had a greater impact on nutrient digestibility. The digestibility of DM (*p* < 0.01), aNDF (*p* < 0.05), starch (*p* < 0.01), and EE (*p* < 0.01) were more affected by FC ratio.

**Table 4:** Chemical composition of feces and diets' apparent digestibility coefficient, divided into four dietary groups (Lsmean ± SE).

**Parameter**	**FC_H (*n.13*)**	**FC_L (*n.13*)**	**LS_no (*n.30*)**	**LS_yes (*n.11*)**
**Chemical composition**				
Dry matter, %	13.80 ± 0.31	13.91 ± 0.50	13.44 ± 0.20^B^	14.64 ± 0.49^A^
Ashes, % DM^−1^	12.01 ± 0.37	11.73 ± 0.39	12.32 ± 0.25	11.58 ± 0.31
Crude protein, % DM^−1^	14.07 ± 0.44	14.02 ± 0.31	13.75 ± 0.20^b^	14.63 ± 0.42^a^
Ether extract, % DM^−1^	1.12 ± 0.04^B^	1.51 ± 0.13^A^	1.19 ± 0.03^B^	1.56 ± 0.14^A^
aNDF, % DM^−1^	56.97 ± 1.04	55.28 ± 1.34	57.16 ± 0.65^a^	53.76 ± 1.45^b^
ADF, % DM^−1^	45.91 ± 0.72^A^	41.09 ± 0.81^B^	44.29 ± 0.53^A^	41.26 ± 1.11^B^
ADL, % DM^−1^	15.88 ± 0.66^A^	13.12 ± 0.45^B^	15.09 ± 0.39	13.54 ± 0.67
Starch, % DM^−1^	1.30 ± 0.19	1.84 ± 0.39	1.24 ± 0.08^B^	2.27 ± 0.43^A^
pH	6.46 ± 0.06^A^	6.17 ± 0.07^B^	6.41 ± 0.04^A^	6.14 ± 0.07^B^
**Apparent digestibility**				
dDM, %	59.87 ± 1.78^A^	52.90 ± 1.45^B^	57.82 ± 1.40	54.78 ± 3.64
dNDF, %	52.19 ± 2.78^a^	45.78 ± 1.49^b^	48.30 ± 1.81	46.07 ± 3.40
dStarch, %	96.17 ± 0.38^A^	93.15 ± 1.48^B^	95.83 ± 0.34	92.07 ± 2.00
dProtein, %	48.62 ± 2.76	46.64 ± 2.33	51.91 ± 2.03	46.01 ± 4.21
dLipid, %	79.99 ± 1.24^A^	69.84 ± 3.25^B^	79.36 ± 1.11	70.39 ± 4.42

FC_H, high forage-to-concentrate ratio (85:15); FC_L, low forage-to-concentrate ratio (65:35); LS_no, diets without linseed; LS_yes, diets with linseed; DM, Dry Matter; aNDF, neutral detergent fiber; ADF, acid detergent fiber; ADL, lignin acid deterse; dDM, apparent digestibility of DM; dNDF, apparent digestibility of NDF; dStarch, apparent digestibility of starch; dProtein, apparent digestibility of Protein; dLipid, apparent digestibility of Lipid. Pairwise comparisons: FC_H vs. FC_L; LS_no vs. LS_yes; ^a, b^*p* < 0.05; ^A, B^*p* < 0.01.

### 3.2 Sequence data analysis and overview of the microbiota composition

The sequencing of the 41 samples generated a total of 6,746,266 reads, with an average of 164,543 ± 29,535 reads/sample. After performing clustering into ASVs and removing chimeric sequences with DEBLUR, the total number of reads was reduced to 2,566,663, with an average of 62,601 ± 11,363 reads/sample. This reduction is expected when applying denoising algorithms such as DEBLUR and reflects the retention of high-confidence biological sequences, thereby reducing the risk of retaining spurious sequences and false-positive variants (37). The processing of the number of reads at each step with DEBLUR is reported in Supplementary Table S2, and the distribution of the number of reads per sample following the DEBLUR procedure is shown in Supplementary Figure S3A. From the ASVs table imported into R, low-frequency ASVs were removed. A total of 15 phyla, 22 classes, 49 orders, 86 families, 196 genera, and 278 species were identified. Subsequently, the low frequencies ASV were removed, reducing the number of taxa to 10 phyla, 13 classes, 28 orders, 51 families, 102 genera, and 120 species. Considering the different sequencing depths of the samples, rarefaction to 10,000 sequences/sample on the dataset agglomerated at species level was used for alpha and beta diversity analyses (Supplementary Figure S3B). During the rarefaction procedure, no samples were eliminated, as all contained at least 10,000 sequences/sample and no species were removed. These 120 species were used to assess alpha and beta diversity. The average percentage abundances of the main taxa (phylum, family, genus, and species) identified across all samples were evaluated to provide a general characterization of the fecal dairy buffalo microbiota.

**Figure 1:**
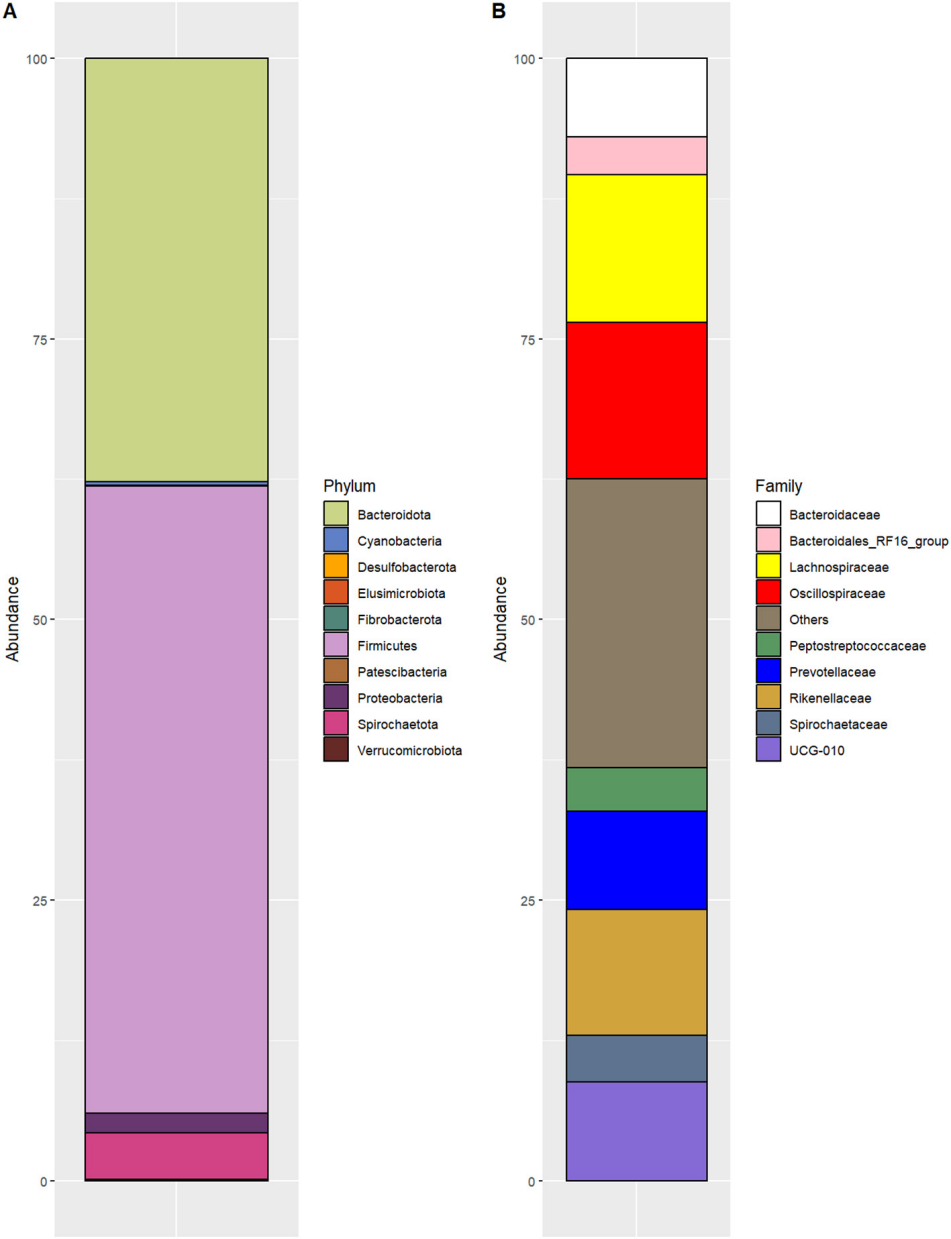
Microbiota characterization of dairy buffalo feces, differential abundance: **(A)** at the phylum level; **(B)** at the family level.

The most abundant phylum (mean ± sd) was *Firmicutes* (55.8 ± 3.6%), followed by *Bacteroidota* (37.7 ± 3.4%), *Spirochaetota* (4.1 ± 1.5%), and *Proteobacteria* (1.8 ± 0.9%; Figure 1A). At the family level, the most abundant taxa were *Oscillospiraceae* (13.9 ± 1.1%), *Lachnospiraceae* (13.2 ± 3.6%), *Rikenellaceae* (11.2 ± 1.3%), *Prevotellaceae* (8.7 ± 1.4%), *Bacteroidaceae* (6.9 ± 0.8%), *Spirochaetaceae* (4.7±1.6%; Figure 1B). Among the most frequently identified genera were an *unclassified Oscillospiraceae* (12.3 ± 1.5%), *Bacteroides* (8.1 ± 0.9%), *Alistipes* (5.6 ± 0.9%), an *unclassified Rikenellaceae* (6.7 ± 0.8%) and *unclassified Prevotellaceae* (3.8 ± 0.6%). A comprehensive list of all taxa identified in the samples is provided in Supplementary Table S3. Finally, the phylogenetic tree of the 120 species, shown in Supplementary Figure S3C, was used for all beta-diversity analyses.

### 3.3 Microbiota characterization by FC ratio

**Figure 2:**
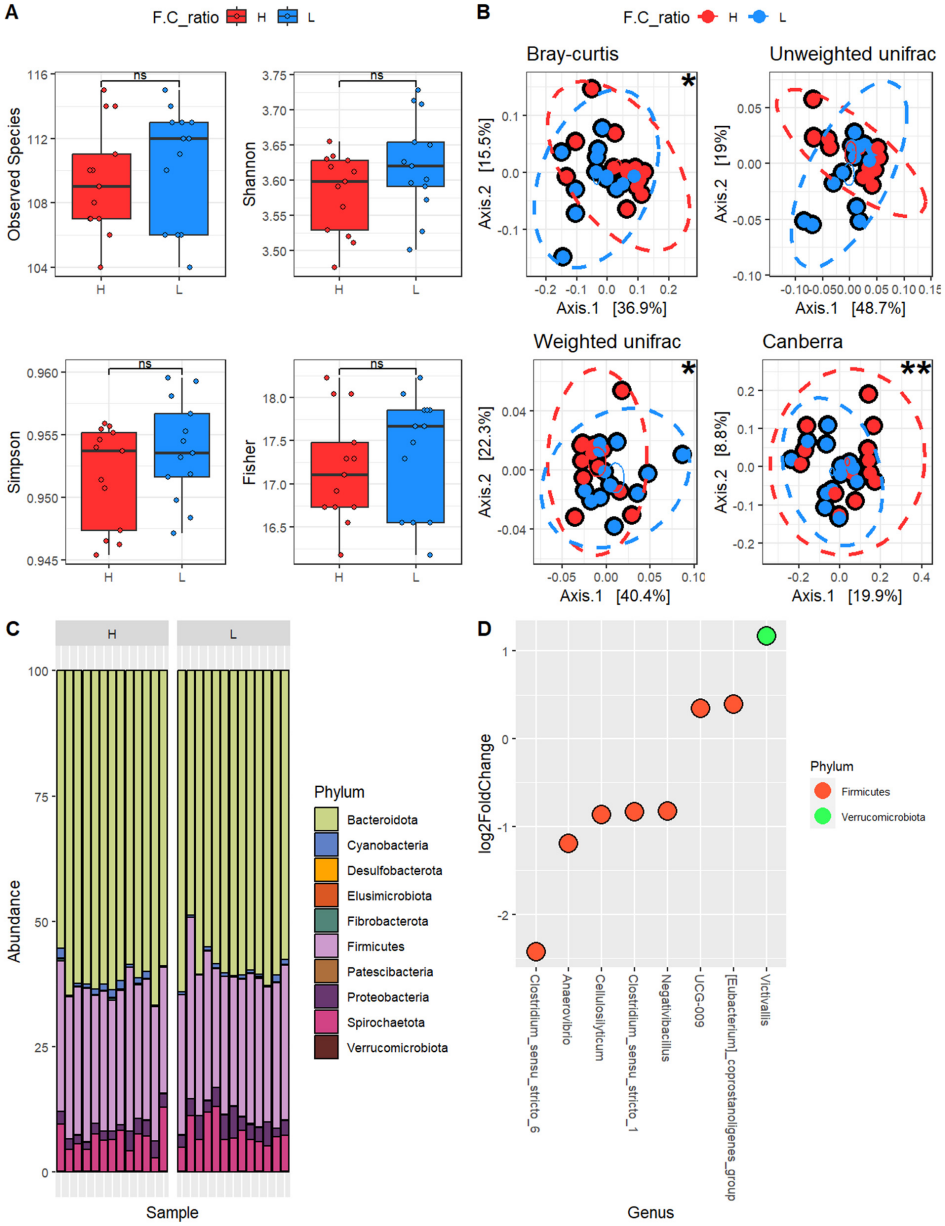
Microbiota characterization based on forage to concentrate (FC) ratio. **(A)** Alpha diversity; **(B)** Beta diversity; **(C)** Abundance of identified phyla, reported for samples with high (FC_H) or low (FC_L) FC ratio; **(D)** Differential abundance at the genus level, where a positive Fold Change indicates that the identified organism is more prevalent in samples with FC_H. Statistical significance is indicated directly in the figure as follows: ns, not statistically significant; □ trend (0.05 <*p* ≤ 0.10); *p ≤ 0.05; **p ≤ 0.01.

Assessments of alpha and beta diversity by FC ratio are shown in Figures 2A, B, respectively. Alpha diversity was assessed using several indices. The Observed Species index reflected the actual number of species, and no significant differences were found for the FC ratio in this case (Wilcoxon-exact test *p*-value = 0.57, Montecarlo permutations = 10,000). The Shannon, Simpson, and Fisher indices, which evaluate both richness and evenness, also showed no significant results for the FC ratio (Wilcoxon-exact test *p*-value for Shannon = 0.22, Simpson = 0.32, Fisher = 0.57, Montecarlo permutations = 10,000). For beta diversity, the Bray–Curtis dissimilarity and Canberra distance were used to evaluate species composition. Bray-Curtis was significantly influenced by the FC ratio using the PERMANOVA test (*p* = 0.01), while Canberra was not statistically influenced by the FC ratio (*p* = 0.12). Additionally, UniFrac distances were used to consider the phylogenetic relationships among all species. Unweighted UniFrac (considering species presence/absence) and Weighted UniFrac (accounting for relative abundances) were employed. The Weighted UniFrac index was influenced by FC ratio (*p* = 0.02) as was the Unweighted UniFrac distance (*p* = 0.005). These results indicate that the FC ratio did not affect alpha diversity (within-sample richness and evenness) but significantly influenced beta diversity, altering the overall microbial community structure between groups.

The next step involved characterizing differentially abundant taxa at the phylum, family, genus, and species levels. All results are reported in Supplementary Table S4 and were evaluated on the entire (unrarefied) dataset normalized with DESeq2. A total of 10 phyla were identified (Figure 2C). The most abundant phylum was *Firmicutes* (55.0 vs. 56.8% in FC_H and FC_L, respectively), followed by *Bacteroidota* (39.3 vs. 37.1%), *Spirochaetota* (3.7 vs. 3.9%), *Proteobacteria* (1.2 vs. 1.7%), *Cyanobacteria* (0.5 vs. 0.3%), *Verrucomicrobiota* (0.2 vs. 0.1%), *Fibrobacterota* (0.05 vs. 0.03%), *Patescibacteria* (0.02 vs. 0.03%), *Desulfobacterota* (0.02 vs. 0.01%), and *Elusimicrobiota* (0.02 vs. 0.01%). There were no significant differences at the phylum level for FC ratio (*p* > 0.05).

Regarding the identified families, statistical differences were observed based on the FC ratio. Specifically, FC_H diets had a suppressive effect on *Lachnospiraceae* (10.9 vs. 14.7% *p* < 0.01, in FC_H and FC_L respectively), *Clostridiaceae* (1.2 vs. 2.3%, *p* < 0.01), *Selenomonadaceae* (0.06 vs. 0.13%, *p* < 0.05) and *Succinivibrionaceae* (0.8 vs. 1.3%, *p* < 0.05) while it enhanced *[Eubacterium]_coprostanoligenes_*group (3.2 vs. 2.5%, *p* < 0.05).

Finally, differential analysis at the genus level was conducted, with results showed in Figure 2D. The FC ratio affected 10 genera. Specifically, FC_H diets increased the abundance of *[Eubacterium]_coprostanoligenes_group* (3.6 vs. 2.9% *p* < 0.01 in FC_H and FC_L, respectively), *Ruminococcaceae uncultured* (1.3 vs. 1.0%, *p* < 0.05), and *Butyricicoccaceae UCG-009* (1.2% vs. 0.9%, *p* < 0.05), *Victivallis* (0.05 vs. 0.02%, *p* < 0.001). In contrast, a reduction was found for *Clostridium_sensu_stricto_1* (1.3 vs. 2.3%, *p* < 0.001), *Cellulosolyticum* (0.7 vs. 1.4%, *p* < 0.001), *Clostridium_sensu_stricto_6* (0.08 vs. 0.45%, *p* < 0.001) and *Negativibacillus* (0.1 vs. 0.2%, *p* < 0.05).

### 3.4 Microbiota characterization by linseed presence

**Figure 3:**
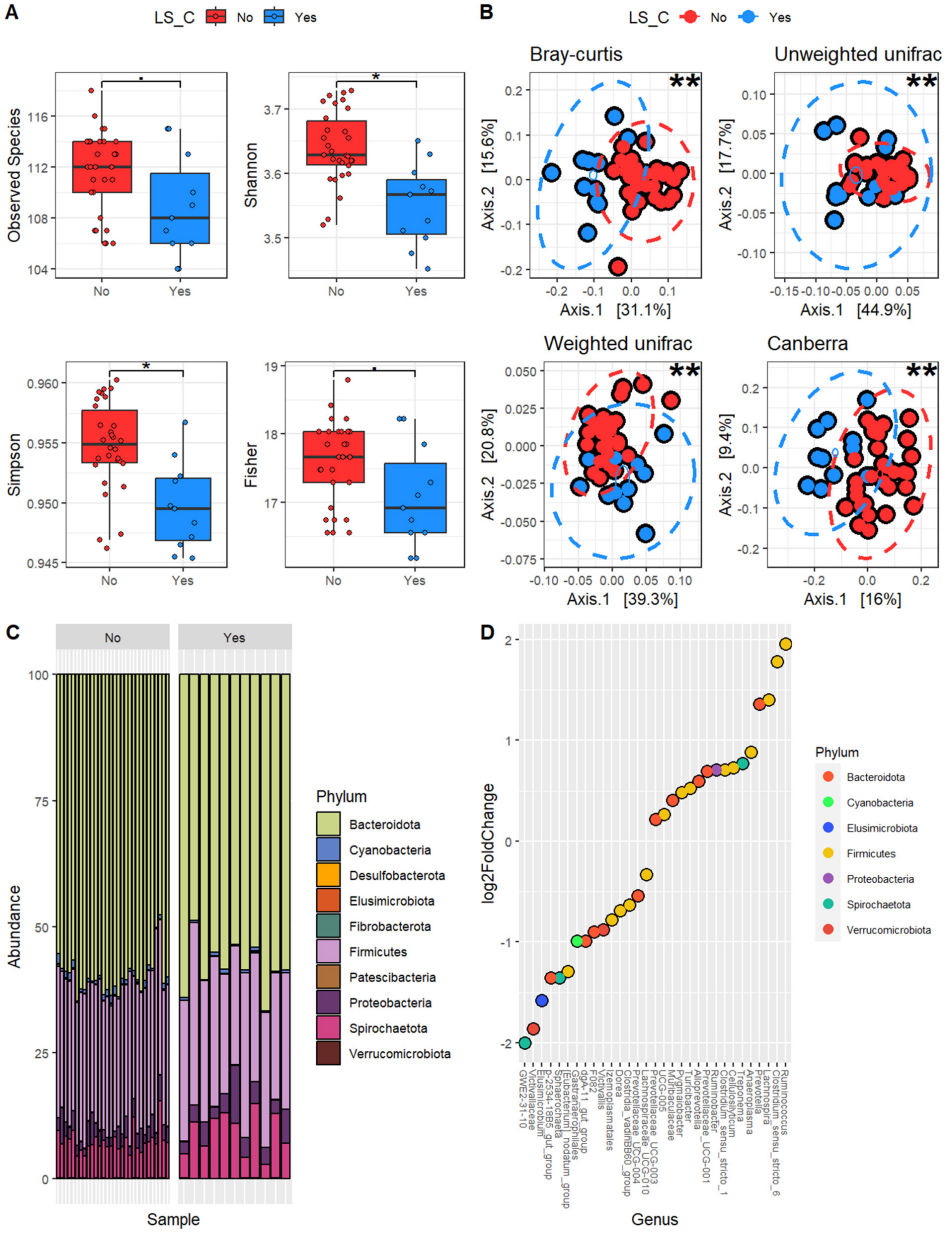
Microbiota characterization based on the presence of linseed (LS). **(A)** Alpha diversity; **(B)** Beta diversity; **(C)** Abundance of identified phyla, reported based on the presence (LS_yes) or absence (LS_no) of Linseed; **(D)** Differential abundance at the genus level, where a positive Fold Change indicates that the identified organism is more prevalent in samples with LS_yes. Statistical significance is indicated directly in the figure as follows: ns, not statistically significant; □ trend (0.05 <*p* ≤ 0.10); *p ≤ 0.05; **p ≤ 0.01.

Alpha diversity (Figure 3A) showed differences between the two groups, with a reduction for all the metrics in LS_yes compared with LS_no. This reduction was significant with the Observed Species index (Wilcoxon-exact test *p* = 0.03, Montecarlo permutations = 10,000), Shannon Index (Wilcoxon-exact test *p* = 0.004, Montecarlo permutations = 10,000), Simpson Index (Wilcoxon-exact test *p* = 0.001, Montecarlo permutations = 10,000), and Fisher Index (Wilcoxon-exact test *p* = 0.05, Montecarlo permutations = 10,000). Beta diversity (Figure 3B) also showed statistically significant differences across all metrics considered: Bray–Curtis (*p* = 0.0001), Canberra (*p* = 0.0001), Weighted UniFrac (*p* = 0.0002), and Unweighted UniFrac (*p* = 0.0002). Consistent with this, greater differences in differential abundance were identified for all analyzed taxa (Supplementary Table S5). For the identified phyla showed in Figure 3C, seven were differentially abundant between the two conditions. Of these, four were reduced in LS_yes diets: *Verrucomicrobiota* (0.15 vs. 0.05%, *p* < 0.001 LS_no and LS_yes, respectively), *Bacteroidota* (38.1 vs. 36.6%, *p* < 0.01), *Elusimicrobiota* (0.025 vs. 0.007%, *p* < 0.05), and *Cyanobacteria* (0.4 vs. 0.2%, *p* < 0.05). The three phyla that increased in the presence of LS were *Spirochaetota* (3.9 vs. 4.6%, *p* < 0.001 LS_no and LS_yes, respectively), *Proteobacteria* (1.7 vs. 2.1%, *p* < 0.001), and *Firmicutes* (55.6 vs. 56.4%, *p* < 0.01).

This difference was also reflected at the family level, with 17 families differentially abundant between the two conditions, and at the genus level, with 34 taxa differentially abundant (Figure 3D). Specifically, in LS_yes diets an increased abundance was observed for *Oscillospiraceae* (13.7 vs. 14.5%, *p* < 0.001 in LS_no and LS_yes, respectively), *Prevotellaceae* (8.4 vs. 9.8%, *p* < 0.001), *Muribaculaceae* (1.8 vs. 2.3%, *p* < 0.001) and *Clostridiaceae* (1.5 vs. 2.5%, *p* < 0.001) and *Erysipelotrichaceae* (1.2 vs. 1.5%, *p* < 0.001). Meanwhile, the presence of LS favored the reduction of *Uncultured Bacteroidota* (2.45 vs. 1.78%, *p* < 0.001), *p-2534-18B5_gut_group* (1.4 vs. 0.5%, *p* < 0.001), and *Victivallaceae* (0.2 vs. 0.05%, *p* < 0.001).

At genus level, LS_yes had a significantly greater abundance of *Prevotella* (0.3 vs. 0.8%, *p* < 0.001, in LS_no and LS_yes, respectively), *Ruminococcus* (0.1 vs. 0.5%, *p* < 0.001), *Clostridium_sensu_strictu 1* (1.6 vs. 2.5%, *p* < 0.001), *Clostridium_sensu_strictu 6* (0.1 vs. 0.5%, *p* < 0.001), *Alloprevotella* (2.4 vs. 3.5%, *p* < 0.001), *Lachnospira* (0.01 vs. 0.03%, *p* < 0.001), *Cellulosilyticum* (0.9 vs. 1.5%, *p* < 0.001) and *Ruminobacter* (1.6 vs. 1.1%, *p* < 0.05). Additionally, several unclassified bacteria from the *Prevotellaceae* family were found to be more abundant in the LS_yes diets. On the other hand, there was a reduction in the abundance of certain genera, such as *Dorea* (0.1 vs. 0.06%, *p* < 0.05), *Elusimicrobium* (0.03 vs. 0.008%, *p* < 0.05), and *Victivallis* (0.05 vs. 0.02%, *p* < 0.05) in the LS_yes.

## 4 Discussion

The aim of this study was to characterize the microbial community of the feces of Italian Mediterranean dairy buffaloes and to identify which dietary factor had the greatest influence on the fecal microbiota. The findings revealed that the FC ratio and LS supplementation are the primary dietary factors affecting the microbiota community. The chemical composition of the diets was similar to that reported in recent studies on dairy buffaloes only in terms of DM and ash content (38, 39). Only fiber content was higher in this study compared with previous studies. Additionally, the physical characteristics varied compared to other studies (38, 39). Clearly, these differences can be attributed to variations in the raw feed ingredients used in the diets.

The diets varied between the considered groups. As we expected, FC_L resulted in a higher MFU and had a lower DM content, higher CP, and higher bottom compared with FC_H. This is attributed to the average presence of by-products (threshers) and the inclusion of green forage, which have high moisture and protein content. Meanwhile, the presence of LS does not affect the chemical composition of the diet, except for a decrease in ash content.

The FC ratio had minimal influence on the FA composition of TMR. In contrast, LS significantly altered the FA profile. In LS_yes diets, there was a notable increase in ALA, while the content of LA decreased compared with LS_no, consistent with findings from previous studies (40). Moreover, the overall content of PUFA was elevated in LS_yes diets, indicating an improved FA profile. Additionally, LS_yes diets led to a significant improvement in the n6 to n3 ratio, reflecting a more favorable balance of essential FA. This modification is crucial, as PUFA are associated with various health benefits for animals (36).

The FC ratio influences apparent digestibility and fecal composition. As reported in the results, FC_L diets led to decreased digestibility across all parameters studied. Several authors (17, 42, 43) have noted that particle size appears to play a critical role in feed digestion mechanisms. Studies on dairy cows (41) found that reducing TMR particle size from 52 to 7 mm improved digestive capacity. However, smaller particle size increases the passage rate and flow of fermentable nutrients from the rumen, potentially impacting hindgut fermentation (42). This finding aligns with the results of the present study, which showed a decline in the digestibility of most components in FC_L diets characterized by a higher proportion of fine particles (>4 mm).

Fecal chemical analysis also revealed lower pH levels in FC_L, suggesting increased fermentation in the gut. This result is consistent with reports indicating that high-concentrate diets, particularly those rich in starch and sugars, lower rumen pH and impair fiber digestibility while altering cellulolytic bacterial activity (43, 44). As discussed earlier, FC_L, with its higher fine material content, tends to have a reduced particle size. This reduction has been associated with an increased rate of particle escape from the rumen, leaving insufficient time for proper digestion (45). Furthermore, the reduced activity of cellulolytic microbes is not only caused by a lower rumen pH but can also result from increased starch fermentation and substrate competition (46).

### 4.1 Alpha and beta diversity

Alpha diversity quantifies species richness and evenness within habitats, reflecting the variety and distribution of species in ecological communities (47). Changes in nutrient supply and availability can shift community structure and bacterial diversity (48). According to previous studies (6, 13), diet significantly impacts the composition of bacterial communities by providing the necessary substrates for bacterial growth, thereby exerting selective pressure on these communities. In the present analysis, the alpha diversity indicated that neither the direct species count (Observed) nor the estimate of species richness (Fisher's α) showed significant differences related to the FC ratio or LS presence. This suggests that the samples do not exhibit a significant variation in species richness, or that any differences present are not substantial enough to affect overall diversity. Other indices, such as Shannon and Simpson, also did not show significant effects of the FC ratio, confirming that the FC ratio does not influence alpha diversity but rather modifies beta diversity, as discussed below.

However, diversity indices based on species abundance, such as Shannon and Simpson, were higher in the LS_no compared to the LS_yes, suggesting that the absence of LS contributes to greater overall evenness and diversity within those communities. These indices account for both the number of species and their distribution and relative abundance. The greater diversity observed in the LS_no may suggest a more equitable distribution of species, with a wider variety of species coexisting in similar abundances. This implies that no single species is dominating the resources, thereby contributing to increased diversity. Group comparisons were performed using non-parametric and permutation-based statistical approaches, which provide robust inference under non-normal and unbalanced designs. Nevertheless, the unequal group sizes may represent a limitation of the present study, as they could reduce statistical power, particularly for detecting small effect sizes.

The addition of LS in the diet appears to significantly modify the composition and structure of the fecal microbiome compared to the diet without LS. The Canberra index indicates differences in the relative abundances of microbial species between the two groups, suggesting that LS may promote some microbial groups while suppressing others. The significant result with Unweighted UniFrac shows differences in the presence or absence of species, indicating that the phylogenetic composition of the microbiome is significantly different between the two diets. Finally, the Weighted UniFrac index suggests that, in addition to the presence/absence of species, the relative abundance of species has also changed, altering the overall structure of the fecal bacterial community.

Diets with different FC ratios seem to primarily influence the composition and abundance of microbial species rather than their presence/absence. This suggests that dietary changes mainly affect the relative amounts of species in the microbiome, rather than introducing or removing specific species. Therefore, while the Unweighted UniFrac index does not detect significant differences in terms of species presence, other indices suggest that FC ratio may influence the structure and abundance of microbial species in the feces. Research on dairy cows has shown that higher concentrate inclusion in their diet reduces microbial variability in feces, typically accompanied by a decrease in fecal pH, which significantly impacts microbial communities (48, 49).

In this study, feces from the LS_no group were nutritionally less dense, with lower levels of starch, EE, and CP, compared to those from the LS_yes group, and they also exhibited a higher pH. These differences in fecal nutrient content and pH may help explain the greater microbial diversity observed in the LS_no group, as fecal starch has been identified as a key factor influencing microbial diversity (50). Moreover, fecal pH is a critical determinant of microbial diversity, much like in the rumen, where lower pH levels are strongly associated with reduced species richness and diversity (48). However, starch and pH are not the only factors influencing microbial diversity. Linseed, which is rich in PUFA, may influence the gut microbiota profile either by promoting the lysis and solubilization of bacterial cell membranes, thereby exerting antimicrobial effects by destabilizing these membranes, inhibiting bacterial growth, and potentially reducing overall microbial diversity, or by serving as metabolic substrates for gut bacteria thus influencing the profile of the intestinal microbiota (36). Linseed has been shown to enhance the growth of bacteria associated with propionate and butyrate production, such as members of the *Prevotellaceae* family (51), as highlighted in this study and discussed further below. It has been observed that an increased abundance of *Prevotellaceae* may further reduce microbial diversity by inhibiting competing bacterial groups (52). In addition to its nutrient composition, LS is particulary rich in bioactive compounds, such as lignans and tannins (40), which can selectively modulate the microbiota. Tannins, for instance, have been shown to inhibit various ruminal bacteria, with sensitivity varying even within the same genus (53). This may help explain the reduced microbial diversity observed in the LS_yes diets potentially due to the bioactive compounds present in LS. Specifically, studies on goat rumen microbiota have reported a decline in bacterial diversity following dietary supplementation with LS (54).

The richness and diversity of bacterial species are widely recognized as key indicators of gut microbiota health. A decline in these parameters can impair the microbiota's functionality and resilience, increasing its vulnerability to colonization by external microorganisms, including pathogens (55). Nevertheless, while reduced microbial diversity is often associated with a higher risk of dysbiosis, in some contexts it may also offer advantages—such as enhanced efficiency in nutrient utilization by the host (56).

### 4.2 Relative abundance

Our results reveal that *Firmicutes* and *Bacteroidetes* were the two most dominant phyla, together accounting for over 94% of the total abundance. These findings align with several other studies, including those by Zhao et al. (57) on Murrah and Nili-Ravi buffalo feces, and by Plaizier et al. (48) on dairy cow feces. The FC ratio did not exhibit significant differences at the phylum level, which contrasts with findings reported in other studies in cow (6, 7).

In the present study, a lower relative abundance of *Bacteroidota* and a higher relative abundance of *Firmicutes* and *Proteobacteria* were observed in the FC_L diets, although these differences were not statistically significant, likely due to group-level variability. Previous studies (58) have shown that an increase in *Bacteroidota* is associated with improved digestibility, and we hypothesize that a similar trend might occur in the present study.

Populations of *Bacteroidota*, along with *Fibrobacterota* and *Cyanobacteria*, have been positively correlated with NDF and ADF levels in the diet (59). This suggests a consistent pattern: the bacterial microbiota of ruminants consuming diets high in lignocellulose tends to be dominated by *Bacteroidetes*, in contrast to diets rich in more readily fermentable carbohydrates (59). *Bacteroidetes* are crucial for maintaining gut homeostasis. Bacteria in this phylum encode numerous enzymes for polysaccharide degradation, complementing eukaryotic genomes by breaking down otherwise indigestible carbohydrates and modulating caloric absorption (12). They play a vital role in the gut ecosystem and are linked to host immunity. Dysbiosis of this phylum may contribute to infections, inflammatory bowel disease, or autoimmune disorders (60). Additionally, some studies (48, 58) suggest that *Bacteroidota* are more efficient at degrading structural carbohydrates compared to *Firmicutes*. This efficiency stems from the higher number of glycoside hydrolase and polysaccharide lyase genes per genome in *Bacteroidota*, allowing them to act as key degraders of complex polysaccharides in plant cell walls (61). This may partially explain the lower apparent digestibility observed in the FC_L diets. However, these effects were not clearly evident in the present study, likely because previous studies mentioned specifically increased the proportion of concentrates in the diet, while this study focused on characterizing the microbiota under different dietary conditions.

The three most abundant bacterial families identified were *Oscillospiraceae, Lachnospiraceae*, and *Rikenellaceae*, with relative abundances ranging from 10 to 14.5%. The FC ratio significantly influenced the prevalence of both predominant and less abundant bacterial families. Specifically, *Lachnospiraceae*, known for their role in the fermentation of non-fiber carbohydrates (42), exhibited a marked response to diets rich in concentrates. This finding aligns with previous findings by Rivera-Chacon et al. (8) who reported a higher abundance of *Lachnospiraceae* in high-concentrate diets. Similarly, Zhang et al. (62) observed an increase in *Lachnospiraceae* when dietary grain inclusion rose from 40 to 70%. Furthermore, as reported by Khafipour et al. (55) and Castillo-Lopez et al. (42), both *Succiniovibrionaceae* and *Lachnospiraceae* are more prevalent in environments with higher levels of soluble carbohydrates, and in the feces of cows fed diets containing finer particles. Altering the particle size of forages can affect not only nutrient availability for the host and production performance but also the substrates and metabolic pathways available to microbes (22), thereby modifying the microbial habitat (42). As discussed earlier, the FC_L diets in our study contained a higher proportion of fine particles. We hypothesize that these particles may have rapidly bypassed the rumen, creating a more acidic environment in the feces and promoting the relative abundance of specific families, such as *Lachnospiraceae*. This aligns with the findings of Castillo-Lopez et al. (42), who also reported lower fecal pH in diets with finer particle sizes. Higher inclusion of concentrate in the diet increases the starch content of the digesta, enhancing fermentation in the large intestine and altering the composition of the microbiota in the colon and feces (48). A lower fecal pH, as observed in the present study, suggests a corresponding decrease in pH within the large intestine. This could be associated with increased production of total SCFA, as reported by Rivera-Chacon et al. (8), who emphasized the impact of high-concentrate diets on SCFA accumulation. Additionally, fecal pH has been proposed as a less invasive method to evaluate intestinal acidification in dairy cows fed high-concentrate diets (8). This underscores the potential of fecal pH as a valuable indicator for assessing the effects of dietary interventions on intestinal health and fermentation dynamics.

At the genus level, decreased abundance of *Victivallis, Eubacterium coprostanoligenes_group*, and *UCG-009* in the feces of animals fed a FC_L diet was observed. This contrasts with previous studies that found *Victivallis* to be more abundant in diets with higher concentrate (63). *Clostridium sensu stricto 1*, which was found in higher amounts in the feces of cows under heat stress, is believed to be a pathogen that can compromise intestinal health (64). Metzler-Zebeli et al. (65) reported that *Clostridium* was correlated with the presence of concentrates in the diet. This is consistent with what observed in this study and may support the hypothesis of impaired digestibility and intestinal issues resulting from excessive fermentation of non-structural carbohydrates in the intestine. Although there were similarities between the present study and others at the phylum and family levels, no clear associations were found at the genus level. This can be explained by the fact that microbiota composition is influenced by a variety of factors, including diet formulation, farm management, geographical location, and individual diversity, as reported by Tang et al. (3).

The inclusion of LS in animal diets significantly influenced the composition of the fecal microbiota at the phylum level, as observed in the present study. Guo et al. (36) and Liu et al. (66) reported similar shifts in microbial populations in goat feces and ileum following dietary supplementation with LS. Comparable changes were also observed in the rumen microbiota of goats by Cremonesi et al. (54) and Wang et al. (51) when LS or its oil was included in the diet.

Notably, our findings revealed a higher relative abundance of *Firmicutes, Proteobacteria, Spirochaetota*, and *Elusimicrobiota*, with a concurrent reduction in *Bacteroidota, Cyanobacteria*, and *Verrucomicrobiota* in LS_yes diets. This shift is consistent with the findings of Guo et al. (36), who observed that both LS and its oil increased the abundance of *Firmicutes*, while *Spirochaetota* increased only with LS oil in the colon of cashmere goats. The increased presence of *Proteobacteria* is particularly noteworthy, as this phylum includes many Gram-negative bacteria, some of which are pathogenic and associated with ruminal dysbiosis (36, 66). Guo et al. (36) also reported an increase in *Proteobacteria* in the duodenum and jejunum of goats fed with LS oil supplementation, suggesting that LS oil might increase the risk of pathogenic invasion or interfere with nutrient absorption. This is an important consideration when evaluating the overall health implications of including LS oil in the diet. Another significant finding was the reduced abundance of *Verrucomicrobiota*, a group of predominantly anaerobic organisms essential for maintaining intestinal health, including the regulation of the mucus layer between the intestinal lumen and enterocytes (67). The observed reduction of *Verrucomicrobiota* in LS_yes diets may potentially affect intestinal health, particularly in the posterior intestine, although this remains speculative. Additionally, our observation of a lower fecal pH in the LS_yes diet may suggest an acidic shift in the gastrointestinal tract (GIT), which may be consistent with the onset of intestinal acidosis (65). This decrease in pH could be attributed to the presence of easily fermentable substrates in the diet, such as starch, proteins, and fats, as discussed earlier, as well as bioactive components in linseed, like lignans and tannins. Such a more acidic environment might inhibit the growth of certain bacterial groups, particularly those preferring more alkaline environment, while promoting bacteria adapted to lower pH. This may partially explain the observed decrease in bacteria such as *Dorea, Victivallis*, and *Elusimicrobium*, which may be more sensitive to pH changes; however, this interpretation remains speculative and requires further investigation.

The inclusion of LS significantly altered the relative abundance of certain bacterial families, including *Clostridiaceae, Oscillospiraceae, Prevotellaceae*. The increased presence of *Prevotellaceae* in LS_yes diets align with the findings of Wang et al. (51), who reported similar microbial trends in the rumen of goats.

As mentioned earlier, LS contains lignans. Previous studies (40) have highlighted the ability of *Prevotella* to metabolize lignans, particularly secoisolariciresinol diglucoside, the most abundant lignan in LS, into bioactive compounds such as enterodiol and enterolactone. These metabolites have been associated with potential health-promoting effects, such as modulation of intestinal microbial balance and anti-inflammatory and anticancer activities (40), suggesting that the enrichment of *Prevotella* could contribute positively to the overall gut ecosystem.

In addition to these microbial shifts, LS has been shown to possess antimicrobial activity, likely due to the PUFA it contains, as discussed earlier. The primary PUFA, particularly ALA, have been reported to exert toxic effects on ruminal cellulolytic bacteria (68), which could further explain some of the microbial changes observed in the present study. This underscores the complex interplay between diet, gut microbiota, and the potential health implications of introducing LS or its derivatives into animal feeds. However, further research is needed to fully understand the mechanisms by which LS affects the gut microbiota and its long-term impacts on animal health and productivity.

### 4.3 Limitations of the study

One limitation of our study is that the fecal samples were collected as group samples rather than individual ones. By pooling samples from multiple animals, we may have compromised the ability to capture inter-individual variations in microbial communities. Consequently, individual-specific differences that could provide more detailed insights into the effects of diet or other factors on microbial composition may have been overlooked. Despite this limitation, the consistent treatment of all samples using the same storage protocol helps minimize the risk of introducing bias or artifacts that could affect the overall conclusions. Future studies should consider individual-level sampling to more accurately represent microbiome variability and refine sample storage protocols.

## 5 Conclusion and directions for future research

These pioneering findings provide valuable insights into the structure of the fecal bacterial community in Italian Mediterranean dairy buffaloes, emphasizing the significant impact of dietary composition on microbial diversity, as reported for other ruminants.

In line with findings from goat and cow studies, LS appears to have partially negative effects on gut health, particularly by reducing microbial diversity. Although LS is typically included in diets to enrich products with polyunsaturated fatty acids, our results indicate effects on microbial diversity, without evidence of impaired digestibility. Further studies are needed to confirm potential implications on gut health.

As dairy buffalo diets increasingly mirror those of dairy cows, with a focus on higher concentrate levels, it is essential to consider the implications of this shift on buffalo health and performance. Diets rich in concentrates and fine particles can lead to greater intestinal fermentation, potentially compromising gut health. Moreover, dairy buffaloes have a heightened requirement for coarse fiber compared to other large ruminants. Coarse fiber is critical for maintaining digestive efficiency, supporting gut health, and improving feed digestibility. Enhancing digestibility can not only boost productivity but also reduce the environmental impact of buffalo farming, a sector poised for substantial growth in the coming years. The identification of key microbial families serves as a foundation for future research into their functional roles and the development of strategies to optimize nutritional management. Such strategies have the potential to enhance animal health, productivity, and environmental sustainability in buffalo production systems. While fecal microbiota analysis remains a valuable tool for assessing bacterial composition linked to digestibility, this study found no statistically significant effects of bacterial populations on apparent fiber digestibility. This limitation may stem from the group-level sampling approach rather than individual-level analysis, as well as the descriptive focus of the study, which aimed to characterize the microbiome under varying dietary conditions rather than testing targeted feeding interventions.

## Data Availability

The data presented in the study are deposited in the NCBI Sequence Read Archive (SRA) repository (https://www.ncbi.nlm.nih.gov/sra), Bioproject accession number PRJNA1356347, link: https://www.ncbi.nlm.nih.gov/bioproject/PRJNA1356347.
