# Supplementary material for: Evidence for modulation of the fecal microbiota profile by diet in lactating buffalo
Source: Front Vet Sci. 2026 Feb 17;13:1739986. doi: 10.3389/fvets.2026.1739986 (PMC12955644; doi:10.3389/fvets.2026.1739986)
Supplement: Supplementary file 1 [file Data_Sheet_1.zip › supplementary files/Supplementary_Material.docx]

Supplementary Material

| 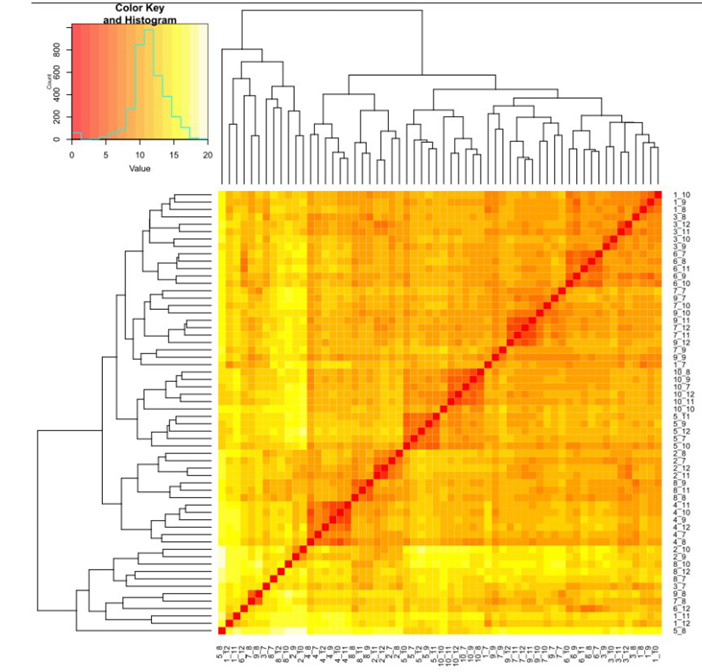 |
| --- |
| **Supplementary Figure 1.** Heatmap of the 60 faeces samples. |


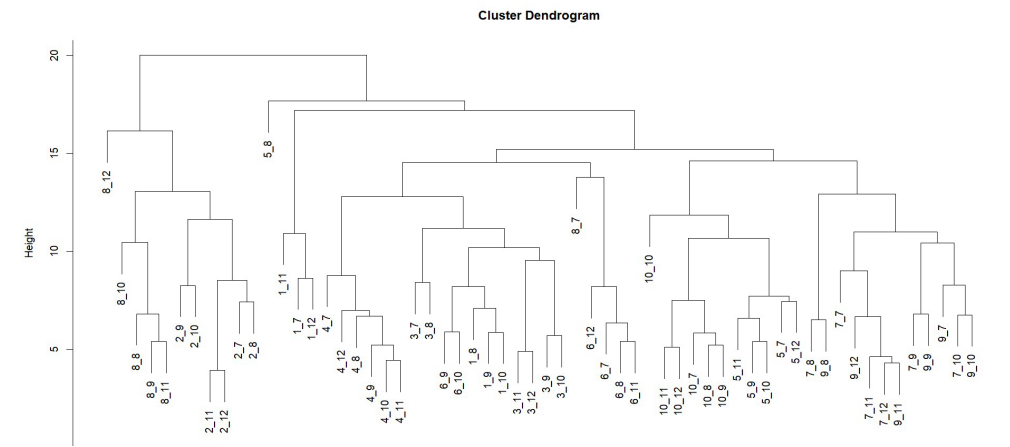


**Supplementary Figure 2.** Dendrogram of the 60 faeces samples.


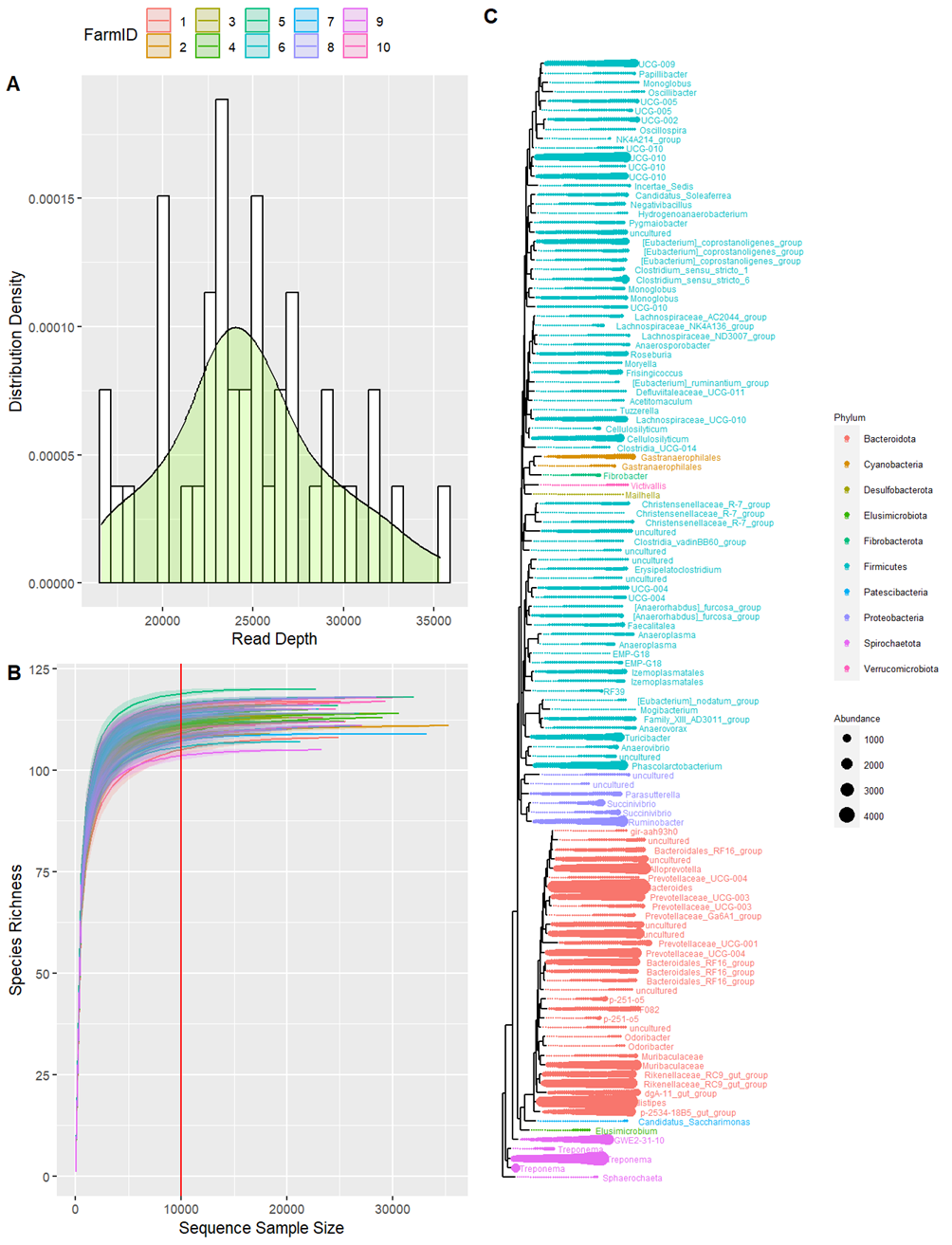


**Supplementary Figure 3.** A) distribution of the number of reads per sample; B) distribution of sequences per sample; C) phylogenetic tree of the 120 species.
